# Prospective Study of Diet Quality and the Risk of Dementia in the Oldest Old

**DOI:** 10.3390/nu15051282

**Published:** 2023-03-04

**Authors:** Ashley C. Flores, Gordon L. Jensen, Diane C. Mitchell, Muzi Na, G. Craig Wood, Christopher D. Still, Xiang Gao

**Affiliations:** 1 Department of Nutritional Sciences, The Pennsylvania State University, State College, PA 16801, USA; 2 Larner College of Medicine, University of Vermont, Burlington, VT 05405, USA; 3 Obesity Institute, Geisinger Health System, Danville, PA 17822, USA; 4 School of Public Health, Institute of Nutrition, Fudan University, Shanghai 200437, China

**Keywords:** diet quality, dietary pattern, oldest old, dementia, aging, prospective study

## Abstract

This study examined the associations between overall diet quality and the risk of dementia in a rural cohort among the oldest old. Included in this prospective cohort study were 2232 participants aged ≥ 80 years and dementia-free at the baseline according to the Geisinger Rural Aging Study (GRAS), a longitudinal cohort in rural Pennsylvania. In 2009, diet quality was assessed by a validated dietary screening tool (DST). Incident cases of dementia during 2009–2021 were identified using diagnosis codes. This approach was validated by a review of electronic health records. Associations between diet quality scores and the incidence of dementia were estimated using the Cox proportional hazards models, adjusted for potential confounders. Across a mean of 6.90 years of follow-up, we identified 408 incident cases of all-cause dementia. Having a higher diet quality was not significantly associated with a lower risk for incidents of all-cause dementia (adjusted HR for the highest compared with the lowest tertile: 1.01, 95% CI: 0.79, 1.29, P-trend = 0.95). Similarly, we did not observe a significant association between diet quality and altered risks of Alzheimer’s disease and other forms of dementia. Overall, having a higher diet quality was not significantly associated with a lower risk of dementia among the oldest old during the full follow-up.

## 1. Introduction

In the United States (U.S.), individuals of advanced ages are expected to rise as the percentage of individuals aged 65 years and over is predicted to increase from 17%, in 2020, to 23%, in 2060, with the oldest old adults will comprise a growing proportion of this population [1]. As the number of older persons in the U.S. population continues to escalate, the risk of dementia, including Alzheimer’s disease, will also increase [2,3]. More than 55 million people were estimated to be living with dementia in 2021, and with projections of 78 million people to be afflicted by 2030, the need for modifiable strategies to reduce the risk of dementia is of high importance [4]. Even though the oldest old is a rapidly growing age group in the U.S. [1] and possesses a greater risk of developing dementia [5], epidemiological research on dementia in this population is limited [6]. Therefore, additional studies to further understand the dementia risk factors in the oldest old age group are warranted.

Targeting modifiable risk factors to prevent dementia has been suggested as an approach to delay or slow down cognitive decline [7]. For instance, nutrition, including adhering to a healthy and balanced diet is recommended for reducing cognitive decline and the risk of dementia [7]. With respect to the oldest old and dementia risk, very few studies have provided insight into the role of individual nutrients or singular dietary components. Although one longitudinal study in this population identified lifestyle factors such as caffeine consumption and supplemental vitamin C intake were linked to reduced dementia risk [8]. Evidence of such associations has been more thoroughly evaluated among other age groups in the literature. For example, prior studies have investigated the associations between the consumption of vegetables and fruits [9,10], meat [11], whole grains [12], and intake of several nutrients [10,13,14] with the risk of dementia among midlife to older adult age groups. It is important to note, the shift from examining singular nutrients and specific food components to dietary patterns as a gauge of diet quality may better explain the role of diet in chronic diseases [15,16]. Prospective studies on the relationship between diet quality or overall dietary patterns on dementia risk, including Alzheimer’s disease have generated inconsistent results, however, these studies are mainly centered on populations of people 60–80 years of age [17,18,19,20,21,22,23,24,25,26,27,28,29,30,31,32,33,34,35]. Limited studies have investigated the influence of diet quality or overall dietary patterns on the subsequent risk of dementia in the oldest old population: aged 80 years and older. Recently, a population-based cohort study, including the oldest old residing in the Varese province, Italy, identified an inverse association between adherence to the Mediterranean diet and dementia prevalence, though, not for incidence [36]. Although granted the promising importance of diet for cognitive health and neurodegenerative disease [37,38,39], the paucity of studies examining the role of diet, particularly overall diet quality, with dementia risk in the oldest old age group highlights the current literature gap among observational studies. By studying the oldest old population, modifiable risk factors may be detected to promote healthy aging, for at-risk, rural populations of advanced age.

We previously demonstrated in our validation analysis of 122 oldest old participants that diet quality scores, as captured by the dietary screening tool (DST), were significantly correlated with scores from the Healthy Eating Index-2015 (HEI-2015) [40]. In our recent prospective cohort analysis of diet quality and Parkinson’s disease in older adults in rural Pennsylvania, we observed higher diet quality was linked to a lower risk of Parkinson’s disease [41]. The results from our studies suggest DST is a valid indicator of diet quality in older adults [42] and the oldest old [40] and warrants further investigations concerning neurodegenerative diseases in populations of advanced age. Thus, the current study aimed to prospectively examine the relationship between diet quality, as assessed by the DST, and the subsequent risk of dementia in a longitudinal rural cohort of the oldest old: aged 80 years and older.

## 2. Methods

### 2.1. Study Population

Beginning in 1994, the Geisinger Rural Aging Study (GRAS) recruited a longitudinal cohort of 21,645 older adults (aged ≥ 65 years) enrolled in a Medicare-managed health maintenance organization per the Geisinger Health System [43]. This cohort included community-dwelling individuals residing in rural central and northeastern Pennsylvania [43]. Representative of rural central Pennsylvania, the GRAS cohort participants are almost entirely non-Hispanic white. Detailed information regarding participant recruitment in the GRAS cohort has been previously described [43].

**Figure 1:**
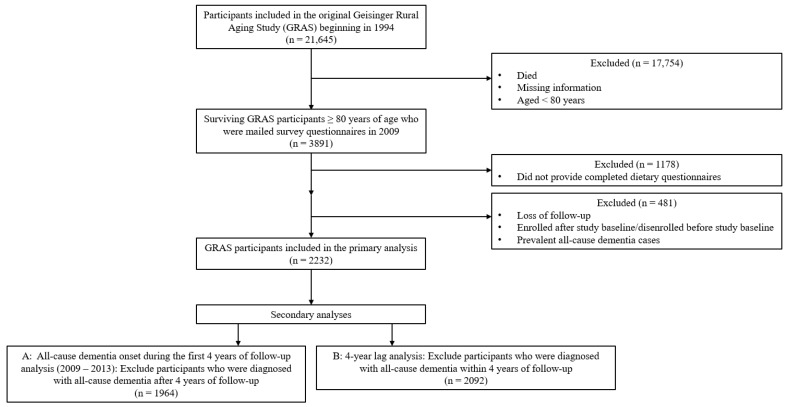
Flow Chart of Geisinger Rural Aging Study (GRAS) Participants. A total of 3891 surviving GRAS participants, 80 years and older, were mailed surveys in 2009. Among participants who received mailed surveys, 2232 participants returned completed dietary information, were not missing during follow-up, were not enrolled after the study baseline or disenrolled before the study baseline based on Electronic Health Record diagnosis data, and did not have prevalent all-cause dementia at baseline. Participants were followed through until 20 July 2021.

In October 2009 (baseline of the study), surveys including health and demographic questionnaires and the DST were mailed to 3891 surviving GRAS participants, ≥ 80 years. Of the individuals who were sent mailed surveys, 2713 participants returned completed dietary information. Participants that did not follow-up were excluded from the analysis (n = 26). We further excluded participants enrolled after the study baseline or disenrolled before the study baseline based on the first and most recent Electronic Health Records (EHR) findings (n = 391). After excluding participants with prevalent all-cause dementia at baseline (n = 64), the 2232 remaining participants were included in the primary analysis (Figure 1). Participants were followed through until 20 July 2021.

The original study conducted in 2009 was approved by the Geisinger Health System Institutional Review Board (Protocol #1999-0112). Approval for participant consent was implied by mailed survey completion. Access to the data used in this study was part of a data use agreement between the Pennsylvania State University and the Geisinger Health System.

### 2.2. Assessment of Diet Quality

To assess diet quality, the DST survey questionnaire comprised 25 questions related to food and behavior associated with dietary intake and was mailed in 2009 to the surviving GRAS participants. The DST was developed using data from multiple 24 h dietary recalls, which were administered to a subsample of the GRAS cohort [44]. The DST questions were derived based on detailed dietary intake data and frequency and temporal distribution analyses of food intake [44]. Cognitive interviewing techniques including concurrent and retrospective methods were used to ensure the population of interest understood the intended concepts [44]. Diet quality was examined with markers of nutritional status and health, including biomarkers, anthropometric, and dietary measures [44]. The total possible score for the DST ranged from 0 to 100 points, with 5 bonus points potentially allotted for dietary supplement uses [42]. An example of a question from the DST is “how often do you usually eat whole grain bread?” To capture intake, participants may select from “never,” “less than once a week”, “1 or 2 times a week”, or “3 or more times a week.” The DST has been validated as a measure of diet quality in older adults [42,44] and the oldest old population [40] in rural Pennsylvania as well as middle-aged adults residing in Appalachia [45]. Further detailed information regarding the DST development and validation has been previously recorded [40,42,44].

### 2.3. Assessment of Incident Cases for All-Cause Dementia

All-cause dementia was the primary outcome in the current analysis. EHR data were utilized to derive an electronic algorithm to identify incident cases of dementia. The following International Classification of Diseases and Related Health Problems, Ninth and Tenth Revision (ICD-9 and ICD-10) codes F01 (vascular dementia), F02 (dementia in other diseases classified elsewhere), F03 (unspecified dementia), and G30 (Alzheimer’s disease) were used.

The accuracy of deriving cases was tested by comparing the results of the identified cases to those revealed by a physician’s review of the individual EHR records. Consultation on the chart review process by a Geisinger neurologist guided two neurology fellowship trainees in the identification of patients with dementia through an independent review of the Geisinger EHR. Included in the review was a randomized sample of 26 GRAS participants with and without codes for diagnosed dementia (identified by ICD 10 codes F01, F02, and F03). The results of the independent reviews were compared and found to have a good agreement (22/26 = 85%, κ = 0.67). The two reviewers re-reviewed the four disagreements and came to a consensus. Diagnostic test measures including sensitivity, specificity, proportions of positive and negative results (PPV and NPV), and accuracy were assessed. If we assume a dementia prevalence of 30%, we find the following diagnostic test measures: sensitivity = 83%, specificity = 88%, PPV = 74%, NPV = 92%, and accuracy = 86%.

### 2.4. Assessment of Covariates

Descriptive information including age, sex, height, weight, educational level, physical activity, diabetes status, hypertension status, coronary heart disease status, living status, living arrangement, antidepressant medication use, and self- or proxy-reporting were obtained at the baseline from the mailed questionnaire data. Information on sex was cross-referenced from the mailed questionnaire data and EHR data. Body mass index (BMI) was determined by weight (kg)/height (m)^2^ and classified as indicated by the National Institutes of Health Guidelines [46]. For BMIs < 18.5 kg/m^2^, participants were categorized as underweight, 18.5–24.9 kg/m^2^ was considered normal, 25.0–29.9 kg/m^2^ was identified as overweight, 30.0–34.9 kg/m^2^ was indicated as obese class I, and the combined obesity classes II and III were set at ≥ 35 kg/m^2^. Demographic information, including race and smoking status, was collected from EHR data.

### 2.5. Statistical Analysis

Baseline descriptive characteristics were presented as means ± standard deviation for continuous variables or as numbers and percentages for categorical variables. Differences between the groups were determined by the Kruskal–Wallis test for non-normally distributed continuous variables, while the chi-square test or Fisher’s exact test was used for the categorical variables.

Cox proportional-hazards models were used to estimate the hazard ratios (HRs) and 95% confidence intervals (CIs) used to test the association between diet quality tertiles and the incidence of all-cause dementia within a 12-year follow-up duration (2009–2021). In all analyses, the lowest tertile in diet quality was used as the reference group. The proportional hazards assumption was tested by adding interaction effects for related covariates by time including age, sex, and diet quality. Since time-dependent sex did not satisfy the proportional hazards assumption (*p*-value < 0.001), sex was included in a strata statement within all Cox proportional-hazards models to permit non-proportionality.

Model 1 was adjusted for age and sex. Model 2 was further adjusted for race, BMI, educational level, smoking status, physical activity, diabetes status, hypertension status, coronary heart disease status, living status, living arrangement, use of antidepressant medication, and self- or proxy-reporting. To test for trends between the tertiles of the diet quality and the subsequent risk for all-cause dementia, we designated participants by the median value of their corresponding tertile in the diet quality as a continuous variable.

To assess the short- and long-term temporal relationship between diet quality and all-cause dementia, Cox proportional-hazards models were calculated for the development of dementia during the first 4 years of the follow-up (2009–2013), and those after 4 years of the follow-up separately. Potential effect modifiers including age, BMI, educational level, and physical activity level for the diet–dementia relationship were assessed by including multiplicated terms in separate models.

All statistical analyses were conducted using SAS version 9.4 (SAS Institute, Cary, NC, USA). Statistical significance was determined at a *p*-value of < 0.05.

## 3. Results

The mean age of the overall cohort was 84.1 years at the study baseline. After a maximum follow-up time of 11.7 years and a mean follow-up time of 6.90 years, 408 cases of incident all-cause dementia were identified. Participants with better adherence to higher diet qualities were more likely to have a higher education level, not ever have been a smoker, and participate in daily physical activities (Table 1). Participants with lower diet qualities were more likely to live alone in either a house, apartment, condominium, or mobile home (Table 1). Additional descriptive or demographic characteristics were not determined to be significant across the tertiles of the diet qualities (Table 1).

**Figure 2:**
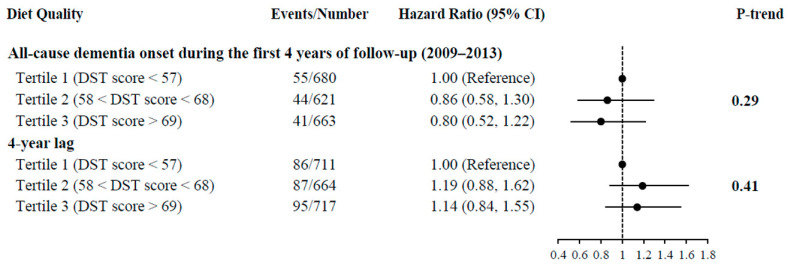
Stratified Analyses by Incidence of All-cause Dementia Onset During the First 4 Years of follow-up (2009–2013) and during the 4-year Lag by Diet Quality Tertile Among Geisinger Rural Aging Study (GRAS) Participants. Abbreviations: CI = confidence intervals; DST = dietary screening tool. Adjusted for age, sex, race, body mass index (BMI), educational level, smoking status, physical activity, diabetes/borderline diabetes, hypertension, coronary heart disease, living status, living arrangement, antidepressant medication, and self- or proxy-reporting. The reference group is Tertile 1 (DST score < 57). Circles indicate hazard ratios with reference to 1.00. Horizontal lines indicate 95% confidence intervals.

Having a higher diet quality was not significantly associated with a lower risk of all-cause dementia (fully adjusted HR for the highest compared with the lowest tertile: 1.01, 95% CI: 0.79, 1.29, P-trend = 0.95) (Table 2). When we limited our analysis to dementia onset during the first 4 years of the follow-up (2009 to 2013), a non-significant trend was demonstrated between having a higher overall diet quality and a reduced risk of developing all-cause dementia (fully adjusted HR for the highest compared with the lowest tertile: 0.80, 95% CI: 0.52, 1.22, P-trend = 0.29) (Figure 2). In contrast, our 4-year lag analysis, excluding participants who were diagnosed with all-cause dementia within the first 4 years of the follow-up, did not materially change the non-significance of the results (fully adjusted HR for the highest compared with the lowest tertile: 1.14, 95% CI: 0.84, 1.55, P-trend = 0.41) (Figure 2).

Furthermore, we did not observe any significant associations between the diet quality and the risk of Alzheimer’s disease (fully adjusted HR for the highest compared with the lowest tertile: 1.04, 95% CI: 0.68, 1.60, P-trend = 0.86) or the risk of other forms of dementia (fully adjusted HR for the highest compared with the lowest tertile: 1.00, 95% CI: 0.74, 1.35, P-trend = 0.99). There were no significant effect modifications between diet quality and potential confounders of interest including age, BMI, educational level, and physical activity (*p*-value > 0.05 for all).

## 4. Discussion

In our prospective cohort study conducted on 2232 oldest old adults in rural Pennsylvania with a mean follow-up time of 6.90 years, overall higher diet quality was not significantly associated with incident all-cause dementia. Similar non-significant associations were observed for different subtypes of dementia. Having a higher diet quality was non-significantly associated with a lower risk of all-cause dementia development during the first 4 years of the follow-up. Therefore, healthy dietary modifications might be suggestive as a protective factor in preventing dementia during short-term follow-up periods, yet may no longer be considered predictive of the incident of dementia risk beyond the 4 years of follow-up.

Several cohort studies have prospectively examined overall dietary patterns or diet quality on the risk of developing dementia, however, the findings are mixed and primarily focused on populations not restricted to the oldest old [17,18,19,20,21,22,23,24,25,26,27,28,29,30,31,32,33,34,35]. Notably, a longitudinal investigation of dietary habits and dementia risk among those 80 years and older did not identify an inverse correlation between greater adherence to the Mediterranean diet pattern, as an indication of diet quality, and a reduced incidence of dementia [36]. This finding is consistent with the results from our study indicating no statistically strong associations between diet quality and the incidence of dementia during the follow-up. To the best of our knowledge, our prospective study is the first to examine the association between overall diet quality as assessed by a screening tool rather than dietary patterns with the longitudinal risk of all-cause dementia among a rural cohort of the oldest old.

Diet, especially dietary patterns, has gained interest as a modifiable factor to encourage healthy aging, however, studies are few among the oldest old [47,48]. Several studies have examined the associations between tea consumption [49], dietary patterns high in red meat, potato, gravy, and butter [50], and dietary diversity [51] with cognitive decline among the oldest old population. Findings pooled together from prospective cohort studies found adherence to better diet quality or a healthy dietary pattern was significantly associated with reduced overall dementia and Alzheimer’s disease risk [37]. Among our GRAS cohort, the oldest old participants reside in primarily rural settings and, therefore, may be more susceptible to disparities that are associated with poor diet quality and unfavorable health outcomes [52]. Older persons, especially the oldest old population, are at risk of having a poor diet and associated malnutrition accompanied by a decline in physiological functions [47]. Likewise, alterations in eating behaviors along with abnormal dietary changes are more often present in individuals with dementia [53,54]. As such, among the oldest old, a decreased quantity of food intake alongside the intake of less varied foods were found to occur more frequently in persons with dementia [36]. We observed a trend between better diet quality and lower dementia risk after restricting our analysis to include incident cases identified within the first 4 years of the follow-up, although not in our 4-year lag analysis. Therefore, more prospective studies with an earlier baseline and longer follow-up duration are warranted.

Our study focused on a rural cohort of the oldest old adults: an age group often understudied in research, where population-based studies with very old age participants are infrequent. Diagnosing dementia in the oldest old can pose several challenges since prospective studies inclusive of this population tend to have smaller sample sizes, in addition to lacking sufficient normative data in the oldest old group [55]. Previous studies on individuals above 85 years of age have found variable results in age-specific incidence rates of dementia and Alzheimer’s disease, thus, implying inconsistencies in determining risk in this age group [56,57]. Further, the age-related likelihood of having mixed dementia increases among community-dwelling older people and the oldest old [58,59,60]. Therefore, we cannot exclude the possibility of other mixed pathologies manifesting among participants diagnosed with Alzheimer’s disease or other dementias in our rural cohort of the oldest old. The results of diet quality and the risk of different subtypes of dementia should be interpreted with caution.

Dementia cases were identified by an electronic search algorithm from EHR data based on ICD codes. This search strategy may be considered less extensive in comparison to a comprehensive manual review, in-person clinical assessments, and validation of each case by a panel of neurologists. In a recent large prospective study of about 500,000 middle-aged to late adults, 1051 cases of total incident dementia, including 352 cases of Alzheimer’s disease, were identified using ICD codes, thus, around 33% of Alzheimer’s disease cases contributed to all-cause dementia cases; however, the potential for under-detecting incident cases of dementia was noted [61]. The reported percentage of Alzheimer’s disease is similar to that observed in our study, although, in contrast to our ascertainment of dementia cases, more extensive ICD codes, including specific subtypes of dementia and general dementia were selected [61]. It may be possible that our search strategy under-identified incident cases and lacked the statistical power required to detect a significant association between diet quality and dementia risk. Although there are limitations to using electronic health records for dementia case detection, such linkage to the Geisinger Healthcare database allows for a streamlined method for identifying cases.

There are several additional limitations to note in our present study. First, we acknowledge we were unable to adjust for energy intake, a covariate often controlled for in analyses of diet quality and other outcomes. Since the DST is limited to questions that were associated with diet quality in the GRAS population, this survey questionnaire may likely be population specific. Therefore, a quantitative assessment of energy or other nutrients is not feasible, although the DST has shown good agreement with HEI scores among the oldest old [40]. A constraint of using electronic medical record data is that each diagnosis was not confirmed in an EHR review by a neurologist, which may lead to the potential for misclassification. Our study population is primarily non-Hispanic white, so our findings may not be generalizable to other populations. Overall diet quality was assessed only at the baseline and, therefore, repeated diet quality assessments may support a better understanding of the development of dementia. Finally, the survey questionnaire relies on self- or proxy-reporting; thus, the results are exposed to recall bias.

Despite the limitations addressed, our study has several strengths. First, our study included a rural population of the oldest old adults, an understudied age group in research. Second, using longitudinal data in our analysis permits an investigation into the long-term relationship between overall diet quality and dementia in an advanced-age population. Our findings add to the limited number of prospective studies regarding this association among the oldest old. Another strength is the use of the DST as a validated measure of diet quality for this population [40]. Lastly, using an electronic algorithm derived from electronic medical record reviews allows for an automated approach that is more efficient and practical for application to large populations.

In conclusion, our findings from our prospective study suggest no associations between overall diet quality and the risk for dementia during the full period of the follow-up. The oldest old population remains an understudied age group, therefore, additional prospective studies with larger sample sizes, earlier baselines, longer follow-up durations, and better ascertainment of dementia and its subtypes are needed.

## Figures and Tables

**Table 1:** Baseline Characteristics Among Geisinger Rural Aging Study (GRAS) Participants by Diet Quality Tertile.

	Diet Quality			
	Tertile 1 (DST Score < 57) (n = 766)	Tertile 2 (58 < DST Score < 68) (n = 708)	Tertile 3 (DST Score > 69) (n = 758)	*p*-Value ^a^
Age, mean (SD), y	84.0 (3.5)	84.1 (3.7)	84.1 (3.5)	0.95
Sex No. (%)				
Men	326 (42.6)	288 (40.7)	305 (40.2)	0.62
Women	440 (57.4)	420 (59.3)	453 (59.8)	
Race No. (%)				
White	761 (99.4)	700 (98.9)	753 (99.3)	0.66
Other	2 (0.3)	1 (0.1)	1 (0.1)	
Unable to obtain or unknown	3 (0.4)	7 (1.0)	4 (0.5)	
Body mass index No. (%), kg/m^2^				
<18.5	17 (2.2)	15 (2.1)	11 (1.5)	0.11
18.5–24.9	215 (28.1)	199 (28.1)	224 (29.6)	
25.0–29.9	292 (38.1)	265 (37.4)	298 (39.3)	
30.0–34.9	126 (16.5)	115 (16.2)	147 (19.4)	
≥35	46 (6.0)	44 (6.2)	27 (3.6)	
Unknown	70 (9.1)	70 (9.9)	51 (6.7)	
Educational level No. (%)				
Below college degree	658 (85.9)	537 (75.9)	543 (71.6)	<0.001
College degree	62 (8.1)	85 (12.0)	133 (17.6)	
Graduate degree	14 (1.8)	35 (4.9)	46 (6.1)	
Unknown	32 (4.2)	51 (7.2)	36 (4.8)	
Smoking status No. (%)				
Never smoker	393 (51.3)	401 (56.6)	461 (60.8)	0.007
Past or current smoker	357 (46.6)	294 (41.5)	284 (37.5)	
Unknown	16 (2.1)	13 (1.8)	13 (1.7)	
Physical activity No. (%)				
Everyday	292 (38.1)	347 (49.0)	464 (61.2)	<0.001
Some days	428 (55.9)	311 (43.9)	258 (34.0)	
Not at all	18 (2.4)	7 (1.0)	4 (0.5)	
Unknown	28 (3.7)	43 (6.1)	32 (4.2)	
Diabetes/borderline diabetes No. (%)				
Yes	184 (24.0)	163 (23.0)	154 (20.3)	0.20
No	582 (76.0)	545 (77.0)	604 (79.7)	
Hypertension No. (%)				
Yes	404 (52.7)	375 (53.0)	424 (55.9)	0.38
No	362 (47.3)	333 (47.0)	334 (44.1)	
Coronary heart disease No. (%)				
Yes	112 (14.6)	108 (15.3)	107 (14.1)	0.83
No	654 (85.4)	600 (84.8)	651 (85.9)	
Living status No. (%)				
Live alone	308 (40.2)	276 (39.0)	296 (39.1)	0.02
With spouse, son/daughter, other family members, or other	441 (57.6)	392 (55.4)	434 (57.3)	
Unknown	17 (2.2)	40 (5.7)	28 (3.7)	
Living arrangement No. (%)				
House, apartment condominium, or mobile home	728 (95.0)	647 (91.4)	711 (93.8)	0.003
Assisted living apartment, care/nursing home, or other	20 (2.6)	15 (2.1)	18 (2.4)	
Unknown	18 (2.4)	46 (6.5)	29 (3.8)	
Antidepressant medication No. (%)				
Yes	72 (9.4)	80 (11.3)	75 (9.9)	0.46
No	694 (90.6)	628 (88.7)	683 (90.1)	
Self- or proxy-report No. (%)				
Self	667 (87.1)	607 (85.7)	676 (89.2)	0.24
Proxy	78 (10.2)	73 (10.3)	64 (8.4)	
Unknown	21 (2.7)	28 (4.0)	18 (2.4)	
Dietary Screening Tool (DST) score, mean (SD)	48.3 (7.3)	63.1 (3.2)	76.0 (5.5)	<0.001

^a^ *p*-values were calculated using the Kruskal–Wallis test for non-normally distributed continuous variables and the chi-square test or Fisher’s exact test was used for categorical variables.

**Table 2:** Incidence of All-cause Dementia by Diet Quality Tertile Among Geisinger Rural Aging Study (GRAS) Participants.

	Diet Quality			
	Tertile 1 (DST Score < 57)	Tertile 2 (58 < DST Score < 68)	Tertile 3 (DST Score > 69)	P-Trend ^a^
	Hazard Ratio (95% CI)			
All-cause dementia onset during full follow-up years (2009–2021)				
Events/Number	141/766	131/708	136/758	
Model 1 ^b^	1.00 (Reference)	1.02 (0.80, 1.29)	0.90 (0.71, 1.13)	0.37
Model 2 ^c^	1.00 (Reference)	1.05 (0.82, 1.34)	1.01 (0.79, 1.29)	0.95

Abbreviations: CI = confidence interval; DST = dietary screening tool. ^a^ P-trend was calculated using the median value of each diet quality tertile as a continuous variable; ^b^ adjusted for age and sex; ^c^ adjusted for age, sex, race, body mass index (BMI), educational level, smoking status, physical activity, diabetes/borderline diabetes, hypertension, coronary heart disease, living status, living arrangement, antidepressant medication, and self- or proxy-reporting.

## Data Availability

De-identified participant data is available upon request made by qualified investigators to the authors.
